# Development and performance simulations of a soft X-ray and XUV split-and-delay unit at beamlines FL23/24 at FLASH2 for time-resolved two-color pump–probe experiments

**DOI:** 10.1107/S160057752400609X

**Published:** 2024-08-05

**Authors:** Matthias Dreimann, Frank Wahlert, Sebastian Roling, Rolf Treusch, Elke Plönjes, Helmut Zacharias

**Affiliations:** a Center for Soft Nanoscience Universität Münster Busso-Peus-Strasse 10 48149Münster Germany; b Physikalisches Institut Universität Münster Wilhelm-Klemm-Strasse 10 48149Münster Germany; c Deutsches Elektronen-Synchrotron DESY Notkestrasse 85 22607Hamburg Germany; University of Tokyo, Japan

**Keywords:** time-resolved pump–probe, XUV, soft X-ray, free-electron laser, two-color experiments

## Abstract

The split-and-delay unit (SDU) at FLASH2 will be upgraded to enable the simultaneous operation of two temporally, spatially and spectrally separated probe beams when the free-electron laser undulators are operated in a two-color scheme. By means of suitable thin filters and an optical grating beam path a wide range of combinations of photon energies in the spectral range from 150 eV to 780 eV can be chosen. In this paper, simulations of the spectral transmission and performance parameters of the filter technique are discussed, along with a monochromator with dispersion compensation presently under construction.

## Abstract 2

A set-up for two-color experiments at FLASH2 utilizing the split-and-delay unit at FL23 and FL24 is presented. It will be achieved by using suitable filters and an additional grating beam path with a dispersion compensated monochromator.

## 1. Introduction

Since the advent of free-electron lasers (FELs) in the extreme ultraviolet (XUV) and soft X-ray spectral region, advanced spectroscopic techniques have been developed which have led to new insights into the electronic processes of atoms (Hikosaka *et al.*, 2010 ▸; Ding *et al.*, 2019 ▸), molecules (Kanter *et al.*, 2011 ▸; Erk *et al.*, 2014 ▸; Amini *et al.*, 2018 ▸), condensed matter (Pietzsch *et al.*, 2008 ▸; Hellmann *et al.*, 2012 ▸; Engel *et al.*, 2021 ▸) and plasma physics (Cho *et al.*, 2012 ▸). The development of variable-gap undulators expanded scientific opportunities as the photon energy dependence of a system can now be investigated with high spectral resolution (Faatz *et al.*, 2016 ▸). This photon energy can be matched to the excitation energy of a system, and fine tuning of the photon energy allows scanning over excitation lines (Faatz *et al.*, 2017 ▸). This enables element-specific experiments and targeting different elements or excitation sites within a molecule. The emergence of two-color FEL schemes, in which two distinct photon energies (colors) are generated from one FEL with a femtosecond or pico­second temporal delay will further expand the scientific opportunities. First experiments were demonstrated at FERMI (De Ninno *et al.*, 2013 ▸), followed by other facilities (Hara *et al.*, 2013 ▸; Prat *et al.*, 2022 ▸). At LCLS a two-color operation was demonstrated which involved two different bunches (Marinelli *et al.*, 2015 ▸). A significant improvement of the tunability was achieved by the variable-gap undulators at FLASH2 (Schneidmiller *et al.*, 2019 ▸).

Pump–probe experiments facilitate access to the temporal evolution of a system. With the first FEL pulse a state is excited site-specifically, while the second pulse probes the status of the system, spectrally matched to the scientific question, and enables observation of the temporal development of the system. Some two-color experiments have already been performed with FEL radiation (Inoue *et al.*, 2016 ▸; Picón *et al.*, 2016 ▸; Pontius *et al.*, 2018 ▸). In general, temporal delays between both colors can be tuned by altering the electron trajectory responsible for the emission of a specific color via chicanes. However, these methods are limited by the present dipole magnet chicanes to sub-picosecond time scales (Hara *et al.*, 2013 ▸; Marinelli *et al.*, 2015 ▸). In addition, spectral modulations appear at the temporal overlap due to interference effects (Allaria *et al.*, 2013 ▸). Recent techniques circumvent the problem of interference, but still limit the delay to below 3 ps (Qin *et al.*, 2017 ▸; Serkez *et al.*, 2020 ▸). For XUV-pump/XUV-probe applications which involve molecular reactions, a longer temporal delay is required (Rath *et al.*, 2014 ▸; Suhasaria *et al.*, 2018 ▸; Zastrau *et al.*, 2014 ▸).

This paper presents an approach to enable such two-color pump–probe experiments with a split-and-delay unit (SDU) at FLASH2 which serves beamlines FL23 and FL24 (Dreimann *et al.*, 2023 ▸). Because the inherent principle of the SDU allows for a spatial and temporal separation, it is the obvious instrument for an additional spectral separation. Nevertheless, a major re-design of its optical concept is required to reach this goal for both pulses, in particular when the spectral separation of both pulses is small. In the present form the SDU relies on total reflection on Ni- and Pt-coated mirrors which do not allow spectral filtering of the provided two-color beam. Spectral filtering can be achieved by the utilization of spectral filters and a monochromator with dispersion compensation, also called a time-delay compensated monochromator, built into one of the two beam paths of the SDU. Examples of this concept have been realized for XUV and soft X-ray radiation (Poletto & Tondello, 2001 ▸; Nugent-Glandorf *et al.*, 2002 ▸; Poletto *et al.*, 2007 ▸; Ito *et al.*, 2010 ▸). To fully compensate for both the angular and the lateral chirp emerging from a grating monochromator requires more than two gratings (Nugent-Glandorf *et al.*, 2002 ▸). But in the XUV and soft X-ray regime significant intensity loss is introduced by reflecting and transmitting on optical elements, and therefore typical set-ups compensate the angular chirp while a minor residual lateral chirp is accepted.

In this paper we present a concept for two-color operation in which one color is singled out by a monochromator. The set-up consists of two cylindrical mirrors and two plane gratings arranged to compensate the angular chirp. In future, with this upgrade the temporal, spatial and spectral separation of the two colors can be achieved. It allows pump–probe studies with two distinct colors. The optical layout, the simulation of the expected operational range, and the performance parameters of this set-up are presented in this paper.

## 2. Design for two-color operation

**Figure 1:**
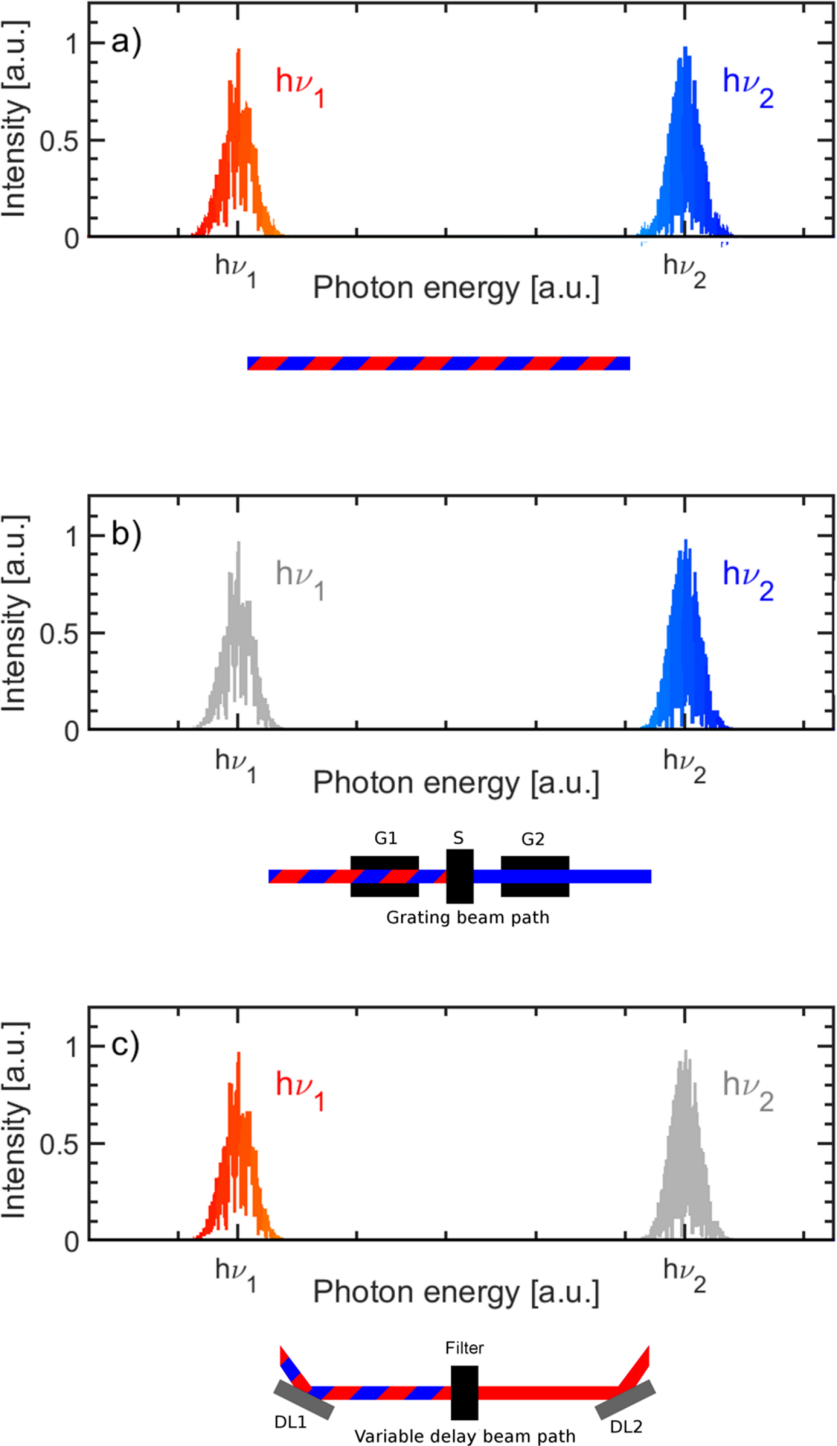
(*a*) Schematic spectrum of a FEL beam in two-color operation. The spectrum consists of two distinct spectral features at *h*ν_1_ and *h*ν_2_ and a spectral gap with a lack of intensity in between. For two-color experiments it is necessary to separate these features for the pump and probe beam. (*b*) Here *h*ν_2_ is singled out in the grating beam path while *h*ν_1_ is blocked. Panel (*c*) depicts the situation where *h*ν_1_ is transmitted in the variable-delay path while *h*ν_2_ is filtered out by an absorbing filter.

At present, FLASH2 offers a photon energy range of 14 eV < *h*ν < 310 eV. The photon energy is altered by means of variable-gap undulators and thereby provides the opportunity of an online photon energy change by a factor of about three (Schreiber *et al.*, 2021 ▸). With a maximum repetition rate of 1 MHz, up to 5000 bunches s^−1^ can be delivered to the user experiment. The typical pulse energy is of the order of 500 µJ and the pulse duration ranges between 200 fs and a few femtoseconds (Schneidmiller *et al.*, 2023 ▸). The possibility to operate a FEL at two different colors which can be tuned up to a factor of three between both colors has been demonstrated at FLASH2 (Schneidmiller *et al.*, 2019 ▸). Therefore, at FLASH2, two-color pump–probe experiments are no longer restricted to the fundamental and its respective harmonics (Pontius *et al.*, 2018 ▸). The two-color operation of the FEL generates X-rays with noticeably different photon energies. However, the colors *h*ν_1_ and *h*ν_2_ are spatially and temporally superimposed within the FEL beam, see Fig. 1 ▸(*a*). In order to perform two-color pump–probe experiments both partial beams must contain only a single color: one beam path, shown in Fig. 1 ▸(*b*), denoted as the fixed delay path, shall only transmit the color *h*ν_2_. It is thus crucial to remove the spectral content *h*ν_1_ from this beam path. For the other beam path, shown in Fig. 1 ▸(*c*), denoted as the variable-beam path, the opposite holds: *h*ν_1_ is transmitted while *h*ν_2_ must be suppressed.

Currently, the SDU allows users to perform time-resolved soft X-ray pump/soft X-ray probe experiments at beamlines FL23 and FL24 at FLASH with sub-femtosecond resolution, as has been described previously (Dreimann *et al.*, 2023 ▸). It allows the users to choose an adjustable temporal delay of −5 ps < Δ*t* < +18 ps with a measured temporal jitter of about *t*_j_ ≃ 120 as. For the grating upgrade the same mechanical structure of the SDU will be employed; therefore we suppose that a similar stability will be achieved also for the two-color operation. The design focus is put on the spectral range from *h*ν = 150 eV to *h*ν = 780 eV which includes the water window as well as *L* shells of magnetic and other elements. At FLASH the spectral range from 700 eV to 890 eV will be available via an afterburner (Tischer *et al.*, 2022 ▸). We consider a set-up with a tunable monochromator as the main spectral selective element and with interchangeable filters as supportive spectral selectors. For the present case the resolving power of the gratings does not need to be high, which is unusual for a monochromator, because the considered photon energies to be separated are already spectrally distinct by typically more than 4% of the photon energy to at least 5 eV. The separation of the two colors is designated to be sufficient when the intensity of the undesired color in one branch is reduced to 1% of the desired color.

## 3. Monochromator with dispersion compensation

An upgrade of the current SDU will allow two-color pump–probe experiments to be performed as it offers the possibility to manipulate both the pump beam and probe beam separately. The present SDU at FLASH2 offers enough space to incorporate an additional beam path with four optical elements. This grating beam path consists of two cylindrical mirrors, two plane variable-line-space (VLS) gratings, and a slit which acts to separate the two colors. The gratings provide spectral dispersion, and the cylindrical mirrors are needed to arrive at the required resolution while minimizing the group delay. These changes are achieved without altering the mechanical parameters of the SDU. Further, the optical path length of each beam should not vary as a function of wavelength, otherwise it would require a determination of the zero delay for every choice of photon energy.

### 3.1. Optical layout

**Figure 2:**
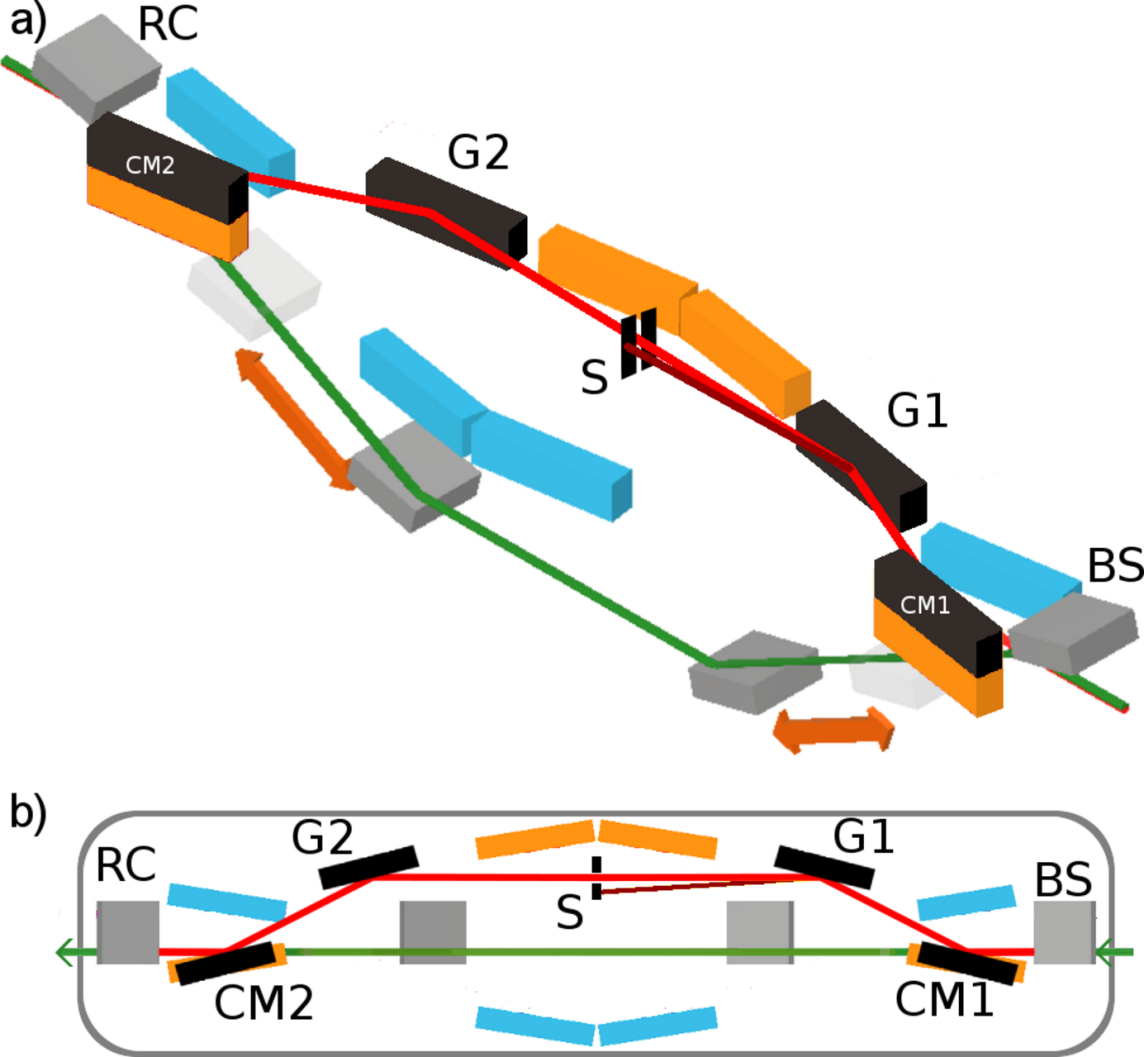
(*a*) Scheme of the grating beam path with mirrors of the variable-delay beam path (gray), grating beam path (black) and both fixed delay beam paths (Ni-coated mirrors: orange; Pt-coated mirrors: blue). (*b*) Top view of the grating beam path. The cylindrical mirror CM1 focuses the beam onto the slit S. The grating G1 is chosen in a Hettrick geometry to CM1, diffracts the beam and forms a focus at the slit S. The grating G2 and cylindrical mirror CM2 form a reversed Hettrick monochromator, thereby compensating the dispersion introduced by the first set-up. The orange mirrors represent the standard Ni-coated beam path. It can be chosen by moving the co-mounted CM1 and CM2 mirrors vertically. The blue mirrors represent the beam path via Pt-coated mirrors for photon energies from *h*ν = 800 eV to *h*ν = 1800 eV which is not affected by the monochromator.

A scheme of the SDU with a new beam path is shown in Fig. 2 ▸(*a*). The corresponding parameters of the optical elements are given in Table 1 ▸. The beam splitting mirror BS spatially separates both beams inside the SDU. One partial beam passes through the variable-delay beam path (beam splitter BS, delay line mirrors DL1 and DL2, recombining mirror RC). When using the monochromator this beam path consists of Ni-coated mirrors. The other partial beam takes the path via two cylindrical mirrors CM1 and CM2 which are Ni-coated, see Fig. 2 ▸(*b*). CM1 then reflects the incoming partial beam onto the Ni-coated VLS grating G1. CM1 and G1 form a Hettrick-type spectrometer (Hettrick & Bowyer, 1983 ▸). The advantage of this set-up is that the imaging properties stay almost constant over a wide range of photon energies. The desired photon energy is selected by tilting the grating. The diffracted beam passes a size-adjustable slit S which is utilized to perform the spectral separation. A grating G2 and cylindrical mirror CM2, arranged in reverse order to CM1 and G1, correct the formerly induced pulse front tilt. The bending radius of the mirror CM2 is chosen such that it restores the original divergence of the incoming beam. Apart from the temporal delay and spectral distribution the beam exhibits the same optical properties after passing through this compensated monochromator. Each partial beam undergoes four reflections. The grating beam path is chosen by moving the co-mounted flat Ni mirror and the Ni-coated cylindrical mirror CM1 mechanically downward.

**Figure 3:**
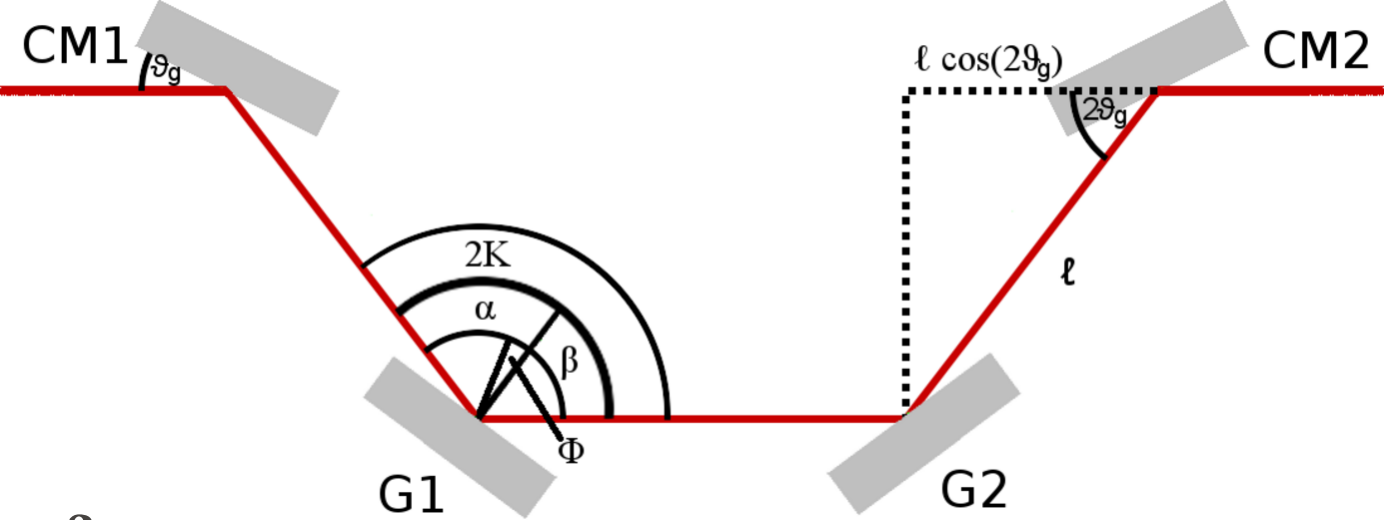
Schematic layout of the grating beam path. The cylindrical mirrors reflect the beam at a glancing angle ϑ_g_. The gratings are described by the angle of incidence α, diffraction angle β, scan angle Φ and constant deviation angle 2*K*.

The temporal delay Δ*t* between both partial beams is given by
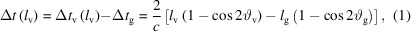
where *l*_v_ = 700–2450 mm denotes the chooseable distance BS/DL1 and RC/DL2 at a glancing angle of ϑ_v_ = 1.8°. The additional grating beam path, see Fig. 3 ▸, is described by the distance *l*_g_ between CM1/CM2 and G1/G2 and the grazing angle of incidence ϑ_g_. As all parameters except *l*_v_ are fixed in the SDU, only *l*_v_ determines the temporal delay between both pulses. For the discussion of the grating beam path, it is more convenient to use the angle of incidence α and diffraction angle β. Both angles are defined from the grating normal to the angle of incidence and diffraction. In this paper α > 0 and β < 0 always applies. For a grating in a Hettrick-type set-up the deviation angle 2*K* = α − β is constant. With the scan angle Φ = α + β, the central grating constant *d*_0_ and the wavelength under consideration λ, the diffracted beam in the order *m* = 1 can be described by
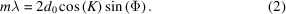
Equation (1) is then modified to
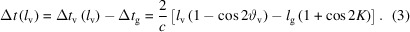
Both gratings are operated in a constant deviation mount, therefore 2*K* remains constant. As the delay of the grating beam path depends on *K*, an adjustment of Φ to the proper wavelength λ according to equation (2) does not change Δ*t*. That means that Δ*t*_g_ is independent of the wavelength that is selected. This has the advantage that no calibration of the temporal overlap of both beams is required when the considered photon energy is changed. An angle of 2*K* = 175° and an optical path length of *l*_g_ = 560 mm result in a delay in the grating beam path of Δ*t*_g_ = 14.22 ps. This value is within 30 fs of the original fixed delay path of Δ*t*_f_ = 14.25 ps, and thus within the typical pulse duration of the FEL. The values for 2*K* and *l*_g_ are chosen in accordance with the space restrictions of the SDU, the stability, the manufacture capabilities of the employed VLS grating and an optimal transmission of the SDU. The nominal temporal delay of the grating beam path should be chosen such that Δ*t*_g_ = 2/*cl*_g_ (1 − cos2ϑ_g_) ≃ Δ*t*_f_ = 14.25 ps, in order to maintain the original delay range provided by the SDU.

### 3.2. Transmission

**Figure 4:**
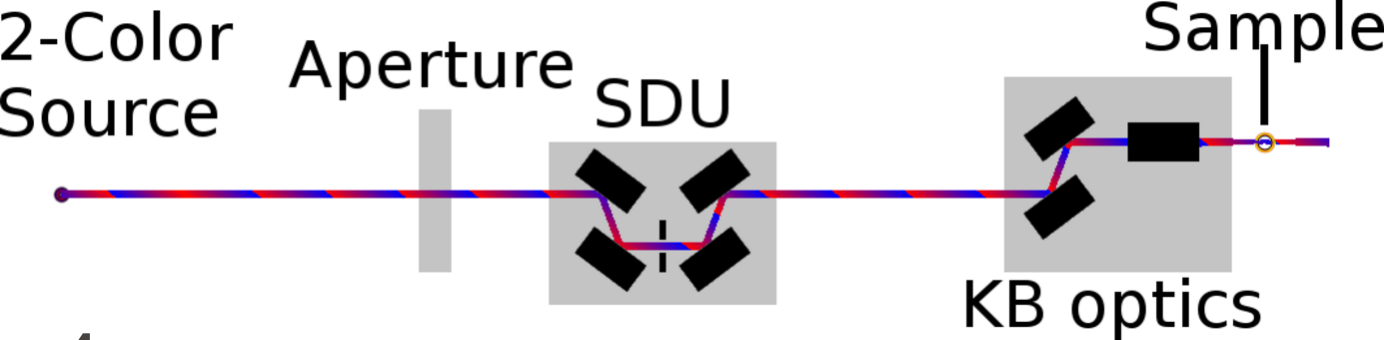
Basic set-up for the simulation of the focal spot size and the group delay. The FEL beam passes a 5 mm aperture at a distance of 48.1 m from the source and is reflected by the first mirror CM1 after an additional 12.7 m. After passing the SDU the beam enters the KB optic KAOS (Manfredda *et al.*, 2022 ▸) which focuses the beam onto the sample. At this sample position the simulation is evaluated. For an extended parameter list see Table 2 ▸.

The expected performance of the monochromator has been evaluated by geometric calculations and the ray-tracing program *RAY-UI* (Baumgärtel *et al.*, 2016 ▸). Calculations of the reflectivity have been carried out via *REFLEC* (Schäfers, 2008 ▸). The set-up for simulation is shown in Fig. 4 ▸. The source is a generated beam which serves as a two-color source. The divergence of the beam is chosen according to Kuhlmann & Plönjes (2013 ▸). At a distance of 48.1 m from the source an aperture of 5 mm diameter confines the beam. The beam then enters the SDU and is monochromated as depicted in Fig. 2 ▸. The first mirror CM1 is located at a distance of 60.8 m behind the source. After passing the SDU the beam is focused onto the position of the sample by a Kirkpatrick–Baez (KB) optic equipped with a bendable mirror system KAOS (Manfredda *et al.*, 2022 ▸). The evaluation of the simulation parameters takes place at a position behind the last mirror of the KB optic of 2.03 m. Both partial beams are evaluated individually to assess their individual contributions.

**Figure 5:**
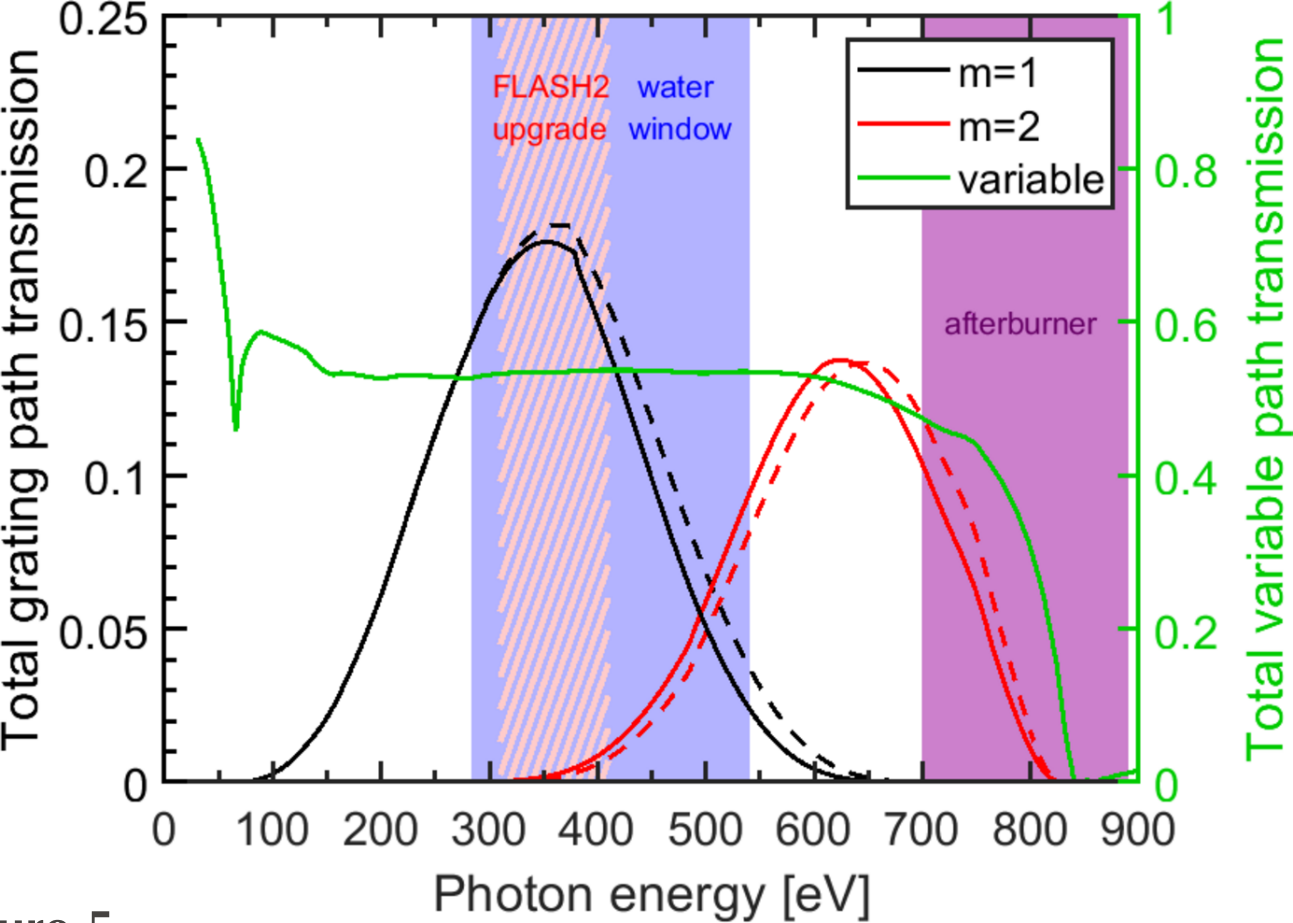
Total transmission as calculated by *REFLEC* (Schäfers, 2008 ▸). Shown is the total transmission of the grating beam path as calculated from actual parameters for the diffraction orders *m* = 1 (solid black line) and *m* = 2 (red line) for γ_G1_ = 0.50° and γ_G2_ = 0.55°. The dashed line shows the reference transmission for a perfect match of both blazing angles at γ = 0.50°. The order *m* = 2 extends into the spectral range of the afterburner (Tischer *et al.*, 2022 ▸). Additionally, the total unfiltered transmission of the variable-delay beam path (green, right vertical scale) is shown. The red hatched area denotes the energy range which will be achieved by the FLASH2020+ upgrade in the near future.

The VLS gratings were produced by the Helmholtz-Zentrum Berlin für Materialien und Energie (HZB) with Si substrates provided by Carl Zeiss SMT, which provided also the Si cylindrical mirrors. The parameters of the optical elements are given in Table 1 ▸. The designed central line density amounts to 200 lines mm^−1^ and at a blaze angle of γ = 0.50°. The optical elements were coated with a 30 nm-thick layer of Ni which provides a relatively uniform spectral reflectivity of about *R* = 0.75 in the considered energy region from 150 eV to about 750 eV. For the grating orders *m* = 1 and *m* = 2 the total transmission of the SDU in the grating beam path is shown in Fig. 5 ▸. The transmission curve is mainly given by the grating transmission. The average blaze angle was measured by the HZB to γ_G1_ = 0.50° and γ_G2_ = 0.55°. In the first diffraction order a maximum transmission of about *T* = 18% is achieved at a photon energy of *h*ν = 350 eV which lies within the photon energy range reachable after the FLASH2 upgrade. The total transmission is decreasing in both directions and amounts to *T* = 10% at *h*ν = 237 eV and *h*ν = 448 eV. Between *h*ν = 156 eV and *h*ν = 538 eV the transmission is larger than 2.5%. The total transmission in the second order increases up to a maximum of *T* = 13.8% at *h*ν = 624 eV and gradually decreases to *T* = 10% at *h*ν = 703 eV and *h*ν = 547 eV. For *m* = 2 the transmission is larger than 2.5% between *h*ν = 783 eV and *h*ν = 449 eV. This configuration still operates with *T* = 1.0% up to *h*ν = 800 eV, decreasing further as the photon energy approaches the Ni *L*-edge. The spectral range of the set-up extends into the envisioned range of the afterburner from *h*ν = 700 eV to *h*ν = 890 eV (Tischer *et al.*, 2022 ▸). The grating beam path thus provides monochromated probe pulses for the water window, the C, N and O *K*-edges, as well as for the *L*-edges of ferromagnetic elements (Cr, Mn, Fe, Co). Due to the slight mismatch of the blaze angles of the two gratings the total transmission of the set-up drops slightly. As shown by the dashed lines in Fig. 5 ▸ for photon energies above *h*ν = 350 eV a difference of the total transmission appears which remains below Δ*T* = 0.02. In the second order the transmission curve is slightly shifted to lower photon energies.

The variable-delay beam path is less complex and therefore the original larger deviation angle of 2*K* = 176.4° is used. Therefore, the total transmission of this beam path stays relative constant at *T* ≃ 0.53 in the considered energy range. For photon energies above *h*ν = 600 eV a slight decrease of the total transmission is prevalent. Still, at *h*ν = 783 eV it amounts to *T* > 36%. This beam path thus provides single-color pump pulses.

**Figure 6:**
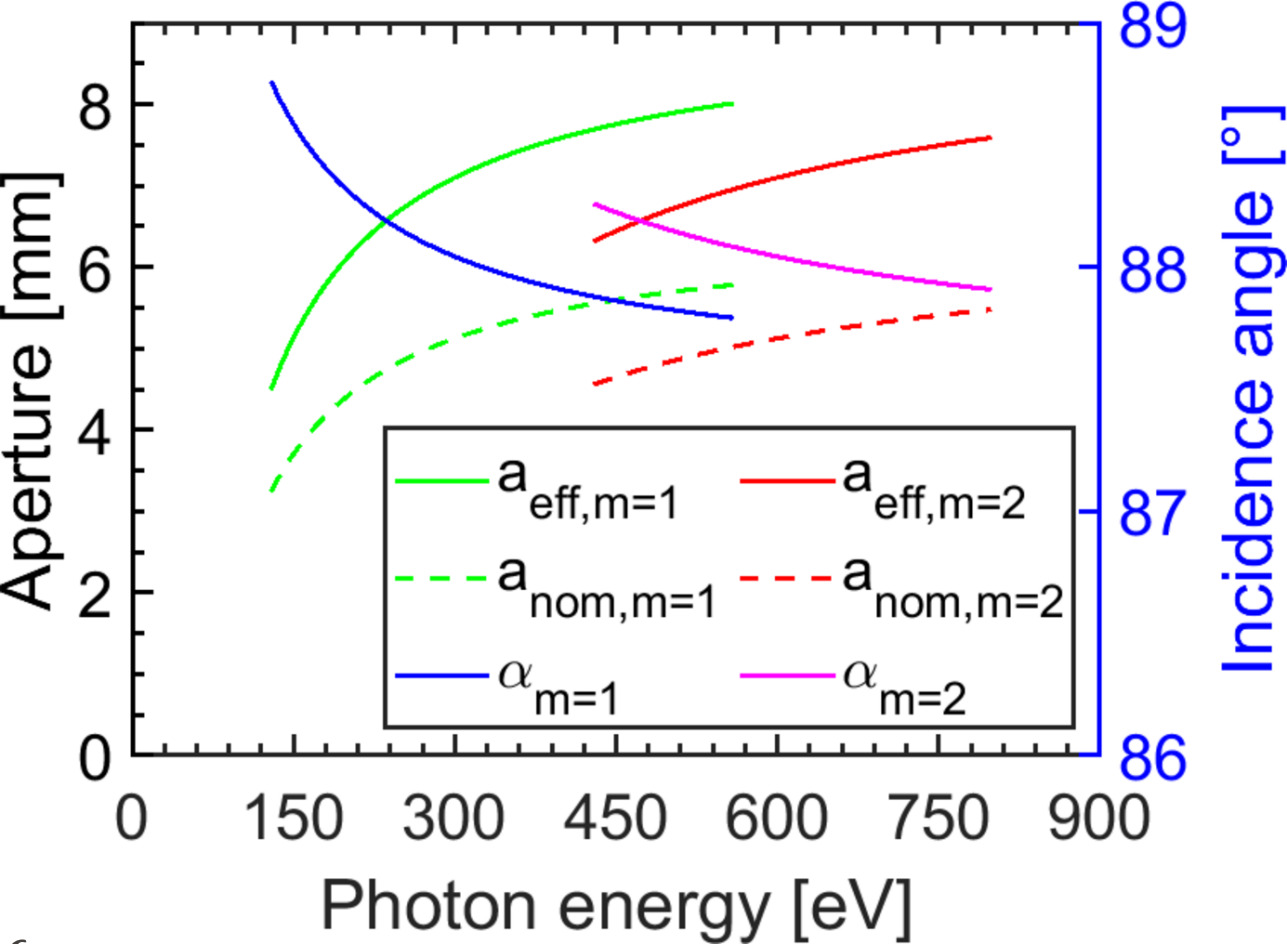
The solid line shows the effective aperture *a*_eff_ of the grating beam path in the first (green line) and second (red line) diffraction order. The focusing property of CM1 increases the nominal aperture *a*_nom_ of the grating (dashed lines) by a factor of 1.38. The nominal aperture is dependent on the angle of incidence on the gratings α (blue and magenta lines) and its clear length of 150 mm.

In fact, the maximal transmission is reduced by the effective aperture of the set-up which is limited by the length of the grating. Fig. 6 ▸ shows the dependence of the effective aperture on the photon energy. For a photon energy of *h*ν = 540 eV the required angle of incidence of α = 87.8° leads to a nominal horizontal aperture of *a*_nom_ = 5.8 mm. Since the mirror CM1 focuses the beam onto the slit S located 1792 mm behind the position of G1, the effective aperture of G1 is increased by a factor of 1.38 to *a*_eff_ = 8.0 mm. α is decreasing with increasing photon energy which results in an increased *a*_eff_. At *h*ν = 350 eV the angle of incidence is α = 88.0°, resulting in an effective aperture of *a*_eff_ = 7.4 mm. Approaching *h*ν = 150 eV the effective aperture is already reduced by the lower angle of incidence of α = 88.6° to *a*_eff_ = 5.1 mm, rapidly decreasing for lower photon energies. In the second order the effective aperture is smaller. Still, in the energy range above *h*ν > 500 eV it amounts to *a*_eff_ > 6.7 mm. It should be noted that for the simulations an upstream aperture of 5 mm diameter is chosen in order that the FEL beam remains smaller than the effective aperture of the grating beam path.

### 3.3. Spectral filtering

**Figure 7:**
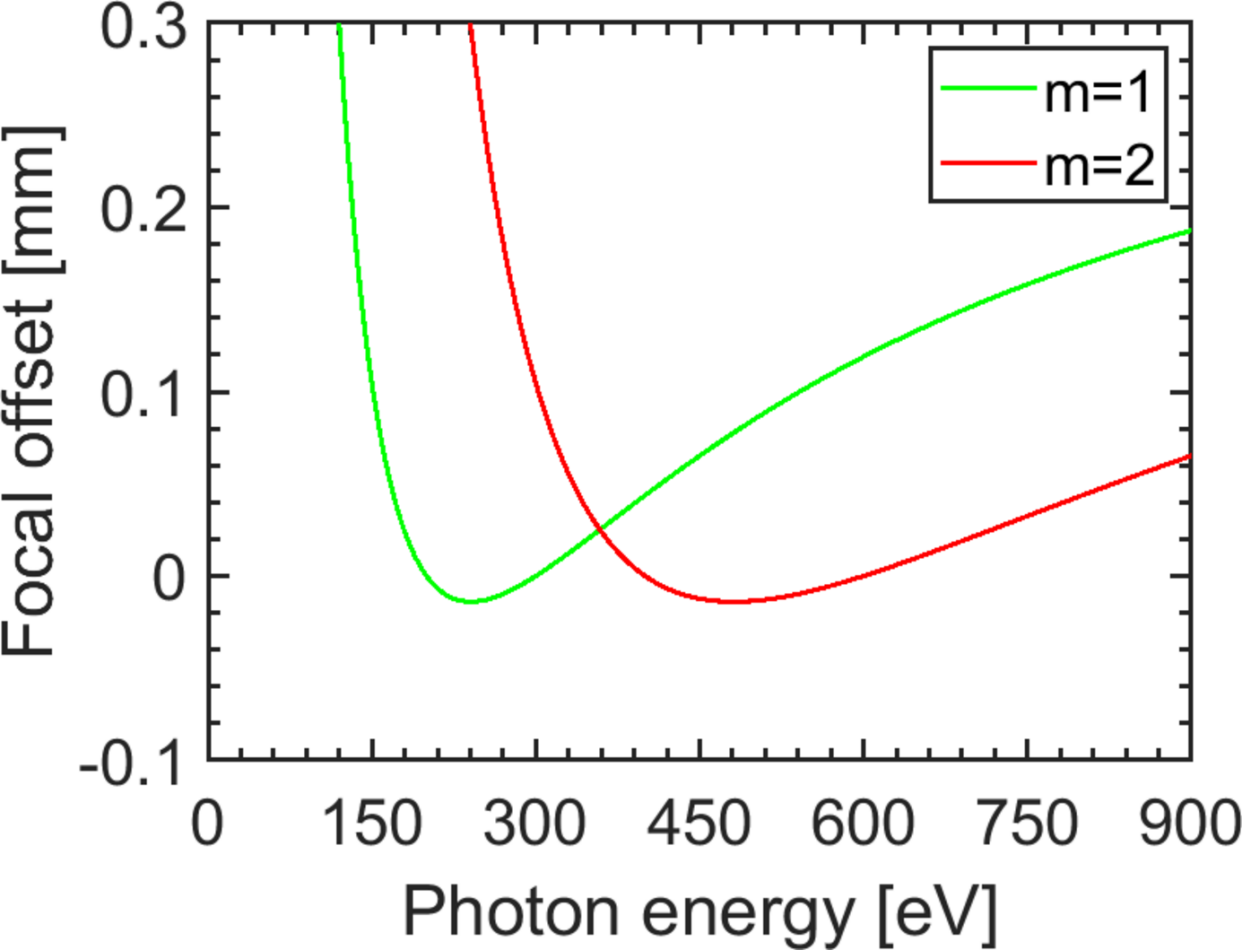
Focal offset of the monochromator along the propagation axis of the beam at the slit in the first (green) and second order (red). The set-up is optimized for photon energies 150 eV < *h*ν < 540 eV. The maximum focal offset in this region amounts to +0.1 mm at *h*ν = 150 eV.

Fig. 7 ▸ shows the calculated nominal focal offset in the propagation direction at the slit position as a function of photon energy. It was optimized for photon energies in the range 150 eV < *h*ν < 540 eV in order to minimize the distortions in this region. The focal offset is −14 µm at a photon energy of *h*ν = 240 eV. For photon energies below *h*ν = 150 eV the focal offset is strongly increasing which leads to a deterioration of the focal overlap of both partial beams after focusing by the KB optic. For photon energies higher than*h*ν = 540 eV an increase of the focal offset occurs in both grating orders but less pronounced in the second order. In the second diffraction order the changes of the focal offset are diminished accordingly by a factor of two. The focal offset of −14 µm occurs at *h*ν = 480 eV.

**Figure 8:**
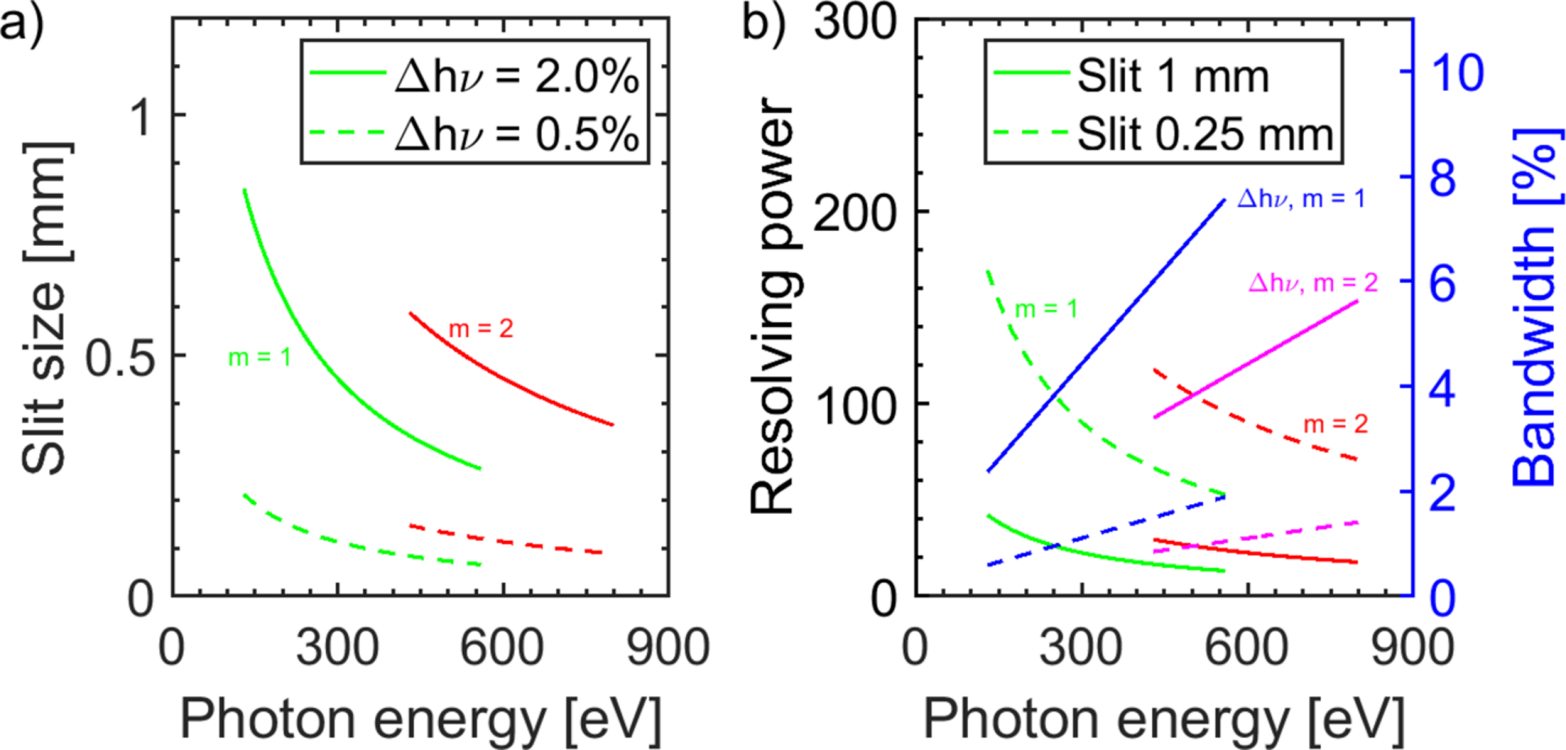
(*a*) Required slit size of the monochromator for the full transmission of a constant bandwidth of Δ*h*ν = 2.0% (solid line) and Δ*h*ν = 0.5% (dashed line) in first (green) and second (red) diffraction order. (*b*) Resolving power *h*ν/Δ*h*ν at a constant slit size of 1 mm (solid line) and 0.25 mm (dashed line) in first (green) and second (red) order. The bandwidth in the first order (blue) increases up to 8% at *h*ν = 600 eV. In the second diffraction order (magenta) a slightly smaller bandwidth is obtained. As described in the text, in this set-up the resolving power is very low compared with typical monochromator systems.

The spectral filtering is carried out via an adjustable slit S1 which defines the resolving power and bandwidth of the system. The bandwidth of the FEL pulse which is around Δ*h*ν ≃ 2% of the photon energy determines the required slit size. For a bandwidth of Δ*h*ν = 2%, corresponding to a resolving power of 50, the slit opening varies from 0.77 mm to 0.27 mm for photon energies from *h*ν = 150 eV to *h*ν = 540 eV, see Fig. 8 ▸(*a*). If angular dispersion is taken into account for a photon energy of *h*ν = 250 eV the focal spot diameter at S1 amounts to 0.66 mm. When a reduced bandwidth is desired, *e.g.* because of a smaller group delay (see below), the slit size has to be reduced accordingly. For a bandwidth of Δ*h*ν = 0.5% this results in a minimum slit opening of 68 µm at *h*ν = 540 eV. The required slit size for a constant bandwidth is somewhat larger in the second diffraction order, see Fig. 8 ▸(*a*). For *h*ν = 700 eV a slit size of 0.4 mm is still sufficient to provide a beam with a bandwidth of Δ*h*ν = 2.0%. In practice, only two colors of the FEL have to be separated. Therefore, when the second color differs by more than Δ*h*ν = 10% from the color to be transmitted, a fixed slit opening of 1 mm is sufficient.

### 3.4. Spot size and temporal broadening

**Figure 9:**
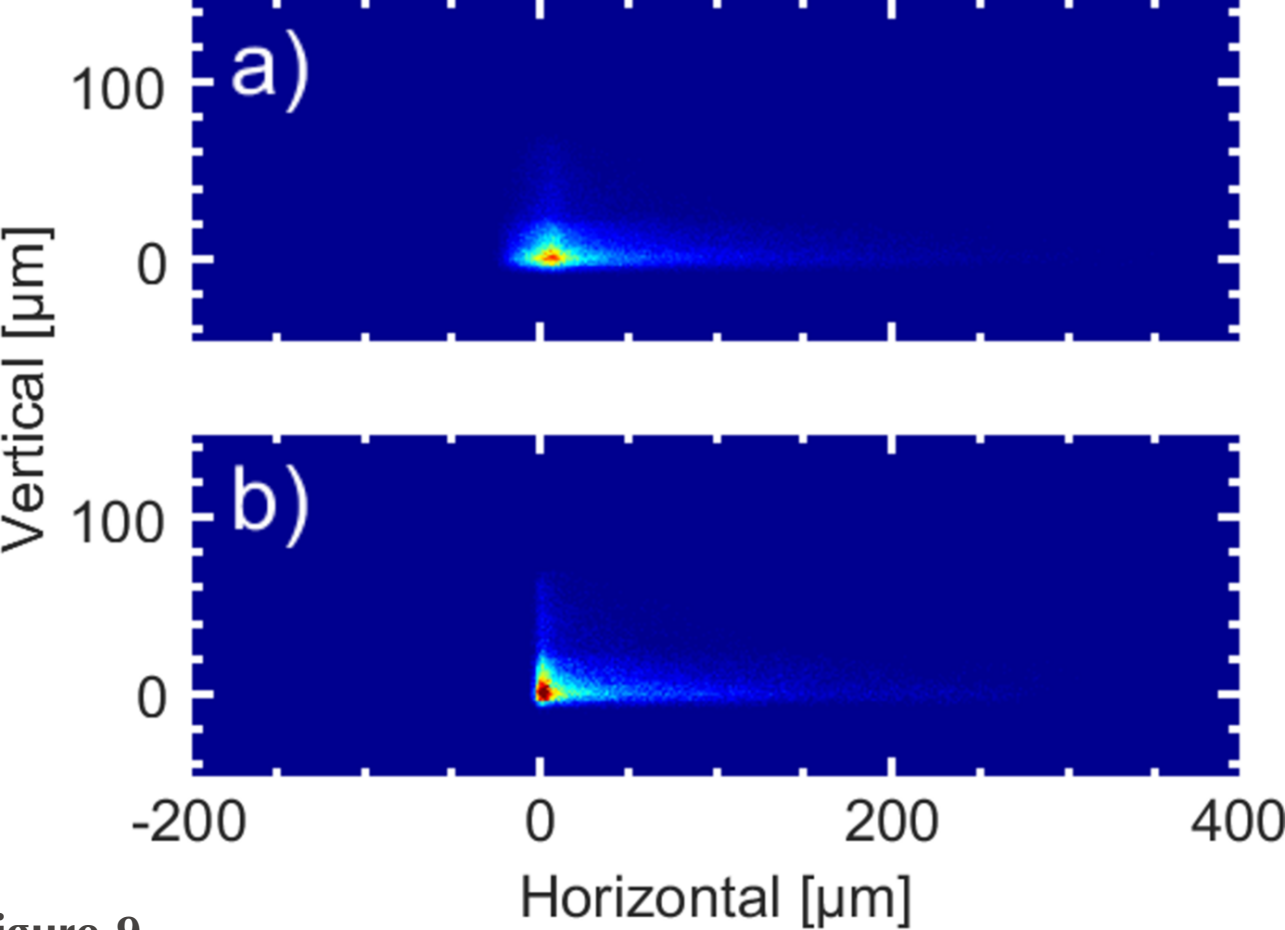
Simulated focal spots at a photon energy of *h*ν = 250 eV at the sample position with ideal mirrors. (*a*) In the grating beam path the simulated spot size amounts to 28 µm (FWHM) in the horizontal and 13 µm in the vertical direction. (*b*) For the variable-delay beam path the focal spot size amounts to 12 µm (FWHM) horizontal and 13 µm (FWHM) vertical. The broader horizontal focus for the grating beam path indicates a residual pulse front tilt from the monochromator set-up.

The spot size of the beam at the experimental interaction point is a crucial parameter for experiments which depend on a high photon intensity. The sizes of both foci are even more important as the two beams are focused via one set of KB optics. Therefore, changing the focus of the beam from the variable-delay beam path directly changes the focus of the beam of the grating beam path, and vice versa. For the grating beam path the simulated spot size at *h*ν = 250 eV and Δ*h*ν = 2% of the grating beam path is shown in Fig. 9 ▸(*a*). The spot size is 28 µm (FWHM) in the horizontal and 13 µm (FWHM) in the vertical direction. A simulation of the variable-delay beam path with identical parameters is shown in Fig. 9 ▸(*b*). In the vertical direction the spot size is still 13 µm (FWHM). This is expected as in the vertical direction no substantial changes of the system occur. On the other hand, in the horizontal direction with 12 µm (FWHM) length the spot size is significantly smaller than for the beam of the grating beam path. Presumably, the broadening is due to a lateral chirp caused by a residual offset of the individual spectral portion when recombining the dispersed spectrum of G1 by its counterpart G2.

**Figure 10:**
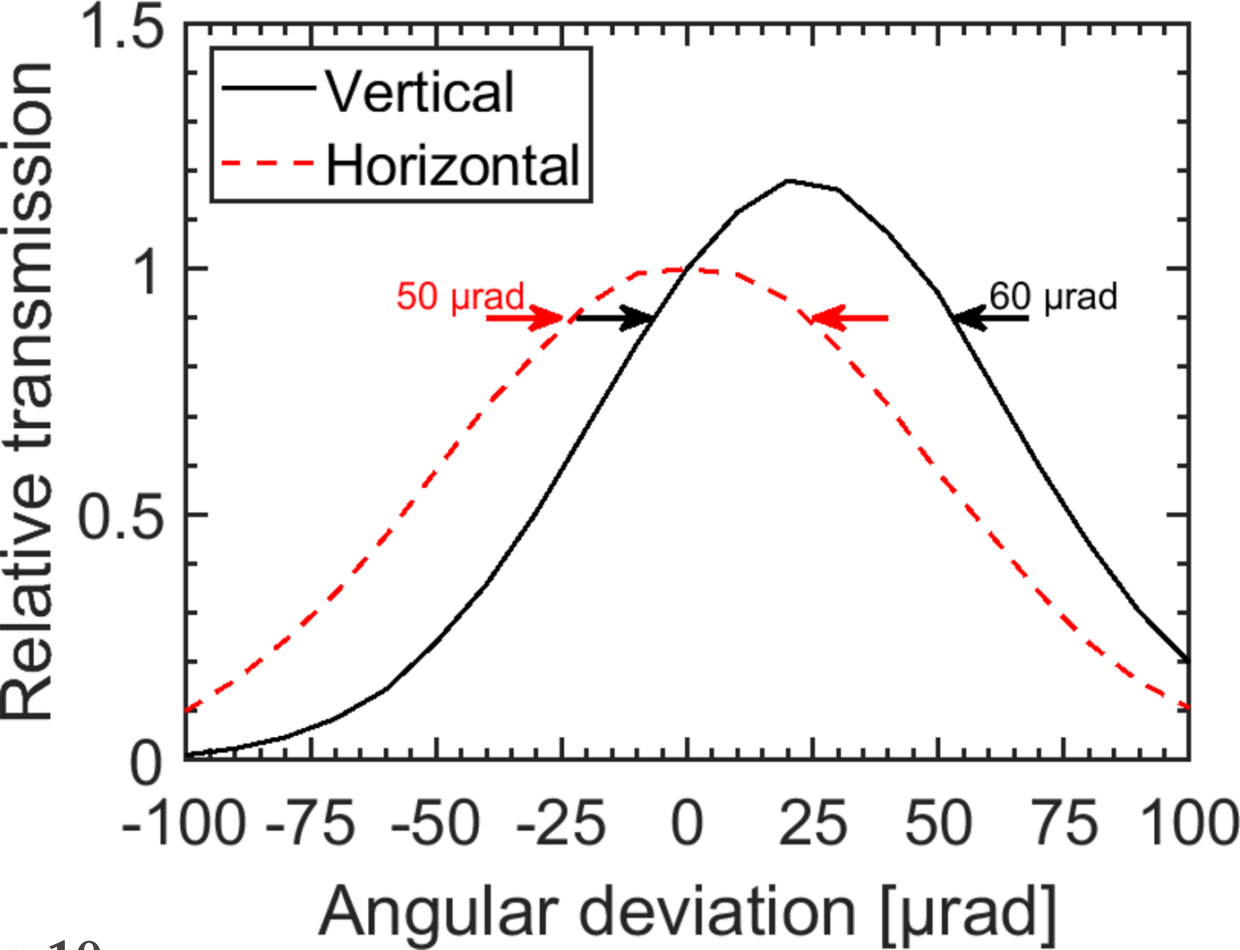
Transmission variation caused by a hypothetical angular deviation at *h*ν = 250 eV. The increase of transmission in the vertical direction (Δ*T* = 118% at Δ*u* = 23 µrad) originates from a greater proportion of the central part of the beam being directed into the grating beam path. A reduction of the intensity to 90% is tolerated by an angular devation of δ*u*_horizontal_ = 50 µrad in the horizontal and δ*u*_vertical_ = 60 µrad in the vertical direction.

The set-up is robust regarding the FEL angular stability *u*. In general, the angular stability *u* can have an effect on the photon flux, see Fig. 10 ▸. However, the properties of the beam remain constant in the range ±50 µrad and strongly degrade for higher angular deviations of the FEL. In the horizontal direction the relative transmission distribution is symmetric. The transmission decreases to 90% within δ*u*_horizontal_ = 50 µrad. In the vertical direction the peak transmission amounts to Δ*T* = 1.18 and is reduced to a nominal 90% within δ*u*_vertical_ = 60 µrad indicating an increased photon flux with an offset of Δ*u* = 23 µrad. This can be explained as the beam is cut by the beam splitter mirror into two halves: when the central part of the beam is directed towards the grating beam path by vertical angular deviation a somewhat higher transmission in this branch arises, while it leads to an equivalent loss of transmission in the variable-delay beam path. Conclusively, the angular deviation of the FEL beam has to be kept below ±50 µrad, that is fulfilled under stable experimental conditions at FLASH2.

**Figure 11:**
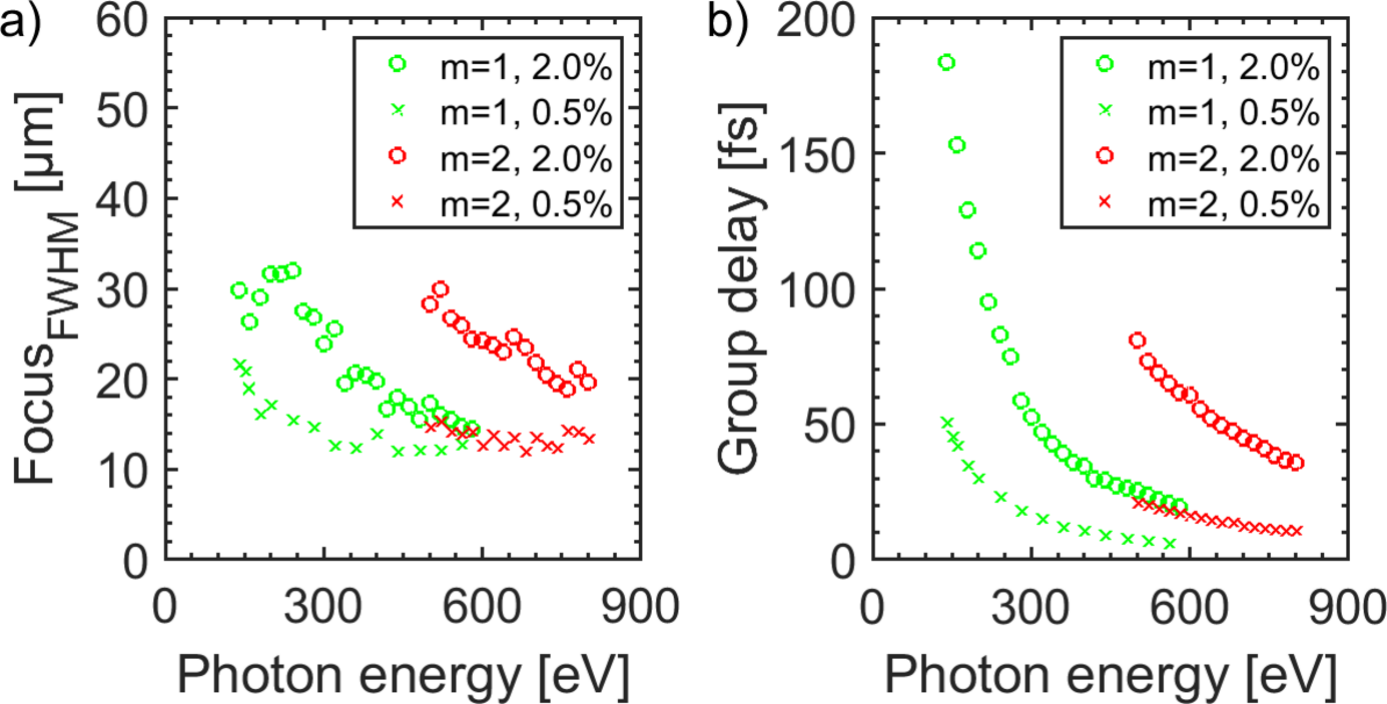
(*a*) Horizontal focal spot size at the sample position. For *m* = 1 and Δ*h*ν = 2.0% (green solid line) the spot size is below 32 µm for *h*ν > 150 eV. For *h*ν = 540 eV the spot size amounts to 15 µm. In this case 19000 lines of the grating are irradiated by light. The focal size decreases for a lower bandwidth of Δ*h*ν = 0.5% (green dashed line). At *h*ν = 150 eV the horizontal spot size is 21 µm where 30000 lines of the grating are illuminated. For *m* = 2 the achievable spot size increases significantly. For Δ*h*ν = 2.0% it ranges between 32 µm and 18 µm. (*b*) At *h*ν = 150 eV the induced group delay of the monochromator set-up is GD_2.0%_ = 160 fs and GD_0.5%_ = 45 fs. With increasing photon energy it decreases significantly. In the order *m* = 2 the group delay has an increased influence on the pulse duration, ranging from GD_2.0%_ = 81 fs at *h*ν = 500 eV to GD_2.0%_ = 36 fs at *h*ν = 800 eV. At *h*ν = 700 eV the group delay amounts to GD_2.0%_ = 45 fs.

Further simulations were carried out in order to examine the spot size variation with respect to the photon energy. The slit size was chosen according to Fig. 8 ▸ providing two different constant bandwidths of Δ*h*ν = 2% and Δ*h*ν = 0.5% at each photon energy. As the dispersive elements are in the horizontal direction, the vertical spot size is not influenced. The results for the horizontal spot size of these simulations are shown in Fig. 11 ▸(*a*). At a photon energy of *h*ν = 140 eV the horizontal spot sizes are Ξ_2.0%_ = 30 µm and Ξ_0.5%_ = 22 µm. This photon energy represents the lower limit as losses due to aberrations increase significantly for lower photon energies. For the smaller bandwidth the spot size is gradually decreasing with increasing photon energy down to Ξ_0.5%_ = 12.4 µm at *h*ν = 540 eV. In contrast, for a greater bandwidth of Δ*h*ν = 2% the spot size first increases to a local maximum of Ξ_2.0%_ = 32 µm at *h*ν = 240 eV and then decreases rapidly for higher photon energies. This is due to the defocusing of the Hettrick set-up in this energy range as shown in Fig. 7 ▸. For a photon energy of *h*ν = 540 eV the horizontal spot size amounts to Ξ_2.0%_ = 15.5 µm. When the set-up operates in the second diffraction order the horizontal spot size is enlarged. At a photon energy of *h*ν = 500 eV it amounts to Ξ_2.0%_ = 28 µm and Ξ_0.5%_ = 14.6 µm. For higher photon energies the spot size decreases gradually until it reaches Ξ_2.0%_ = 20 µm and Ξ_0.5%_ = 13.3 µm at *h*ν = 800 eV.

The associated group delay (GD) within the bandwidth of the set-up is shown in Fig. 11 ▸(*b*). For a bandwidth of Δ*h*ν = 0.5% and *m* = 1 the group delay stays below GD_0.5%_ < 45 fs for all photon energies within the whole spectral working range. For a greater spectral bandwidth the group delay may become significant. For FEL pulses with Δ*h*ν = 2.0% a group delay of GD_2.0%_ < 50 fs holds true for photon energies *h*ν > 300 eV. For lower photon energies the group delay has to be taken into account as it increases strongly. For *h*ν = 150 eV the group delay amounts to GD_2.0%_ = 160 fs so that a reduced temporal resolution has to be considered. For the diffraction order *m* = 2 it is more difficult to achieve low group delays. At a photon energy of *h*ν = 500 eV the associated group delay amounts to GD_2.0%_ = 81 fs. With increasing photon energy it decreases further down to GD_2.0%_ = 36 fs at *h*ν = 800 eV. In general, in the grating beam path the GD can be further linearly reduced when a reducing beam aperture is introduced.

## 4. Optical filters

In a Hettrick-type spectrometer every single optical element has a fixed focal length and is aligned to its designated position (Hettrick & Bowyer, 1983 ▸). A deviation from this position leads to a strong defocusing of the whole set-up. The optical concept of having a variable temporal delay between two partial beams is based on the movement of the two inner optical elements within the beam path. This contradicts a Hettrick set-up. Therefore, the monochromator will be installed only as a beam path with a fixed delay. In the variable-delay beam path the separation of the two colors is achieved via absorption. This is implemented in the SDU in the form of a stage with interchangeable filters. As shown in Fig. 1 ▸, *h*ν_1_ and *h*ν_2_ are filtered in their respective beam paths. Therefore, it is convenient to use the photon energies *h*ν_t_ and *h*ν_a_ which are defined regardless of the beam paths or photon energy. *h*ν_t_ is the photon energy which shall be transmitted by the filter while *h*ν_a_ shall be absorbed within the filter.

At first glance, filtering by means of absorbing gases seems to be a convenient way to remove undesired photon energies in a vacuum environment. As the pressure can be precisely adjusted, they offer a great tunability of the absorption. Yet, the absorption has to be of the order of *A* = 0.99 to 1 to obtain a noticeable filter effect of a color. As an example, using Ar and an absorption length of 1 m, a pressure of 0.1 mbar leads to *A* = 0.05–0.7 for photon energies of the fundamental of FLASH2, which is not satisfying for a standard user operation. Therefore, the most straightforward solution employs thin solid state filters for optical absorption and transmission. In the present set-up these filters can be exchanged and adapted to the specific experimental conditions.

Due to the nature of XUV and soft X-ray photon energies, atomic inner-shell absorptions form the basis of the filtering effect. The quality of the spectral purity of both colors is conveniently defined by the filtering ratio FR,
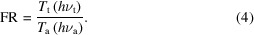
*T*_t_(*h*ν_t_) describes the desired transmission at photon energy *h*ν_t_ and *T*_a_(*h*ν_a_) denotes the tolerable transmission of the undesired color at photon energy *h*ν_a_. That means that for a desired filtering ratio of FR = 100 the intensity of the unwanted color should be only 1% of that of the desired color. The maximal transmission of the desired color is reduced by the required thickness of the filter which has to be chosen such that the intensity of the undesired color is reduced to the appropriate value. Assuming that reflection can be neglected due to a perpendicular angle of incidence, and a refractive index close to 1, the transmission *T*_t_(*h*ν_t_) of a thin filter of thickness *q* is defined by the Beer–Lambert law with extinction coefficient ɛ_0_, density of material ρ and filter thickness *q*,
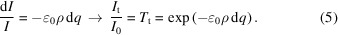
The exponent can be simplified by a reference transmission *T*_R_(*h*ν_t_) = 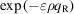 in which *T*_R_(*h*ν_t_) denotes the transmission of a filter of thickness *q*_R_. Thus *q* is measured in units of *q*_R_. It should be kept in mind that *T*_R_(*h*ν_t_) is always below unity due to absorption,
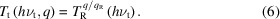
As expected, the transmission decreases with increasing thickness of the filter. Combining equations (6) and (4) results in
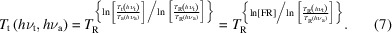
Since *T*_R_ is predetermined by the material of the considered filter of thickness *q*_R_, equation (7) determines the maximum achievable transmission at the photon energy *h*ν_t_ while reducing the pulse energy of the spectral part at *h*ν_a_ by a factor of FR. In equation (7), *q* is determined by *h*ν_t_, *h*ν_a_ and FR, and therefore does not in general remain constant when either of the aforementioned quantities varies,
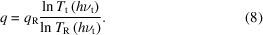
When *T*_R_ is known for all materials eligible as thin filters one can calculate the best filter material for the given task. Besides technical limitations only the quantities *h*ν_t_, *h*ν_a_ and either *T*_t_(*h*ν_t_, *h*ν_a_) or FR, which are part of the experimental parameters, define the filter parameters material and thickness. The measure for the effectiveness of the chosen filter material is either FR when *T*_t_(*h*ν_t_, *h*ν_a_) is specified by the experimental conditions, or vice versa. Obviously, it is generally not possible to specify both *T*_t_(*h*ν_t_, *h*ν_a_) and FR in an experiment. A smaller energetic difference of both photon energies, in general, requires a stronger filtering and consequently thicker filters, which eventually increases the loss for the transmitted photon energy. For *h*ν_t_ ≃ *h*ν_a_ it follows that *T*_t_(*h*ν_t_,*h*ν_a_) ≃ 0 as both colors are no longer spectrally distinct.

**Figure 12:**
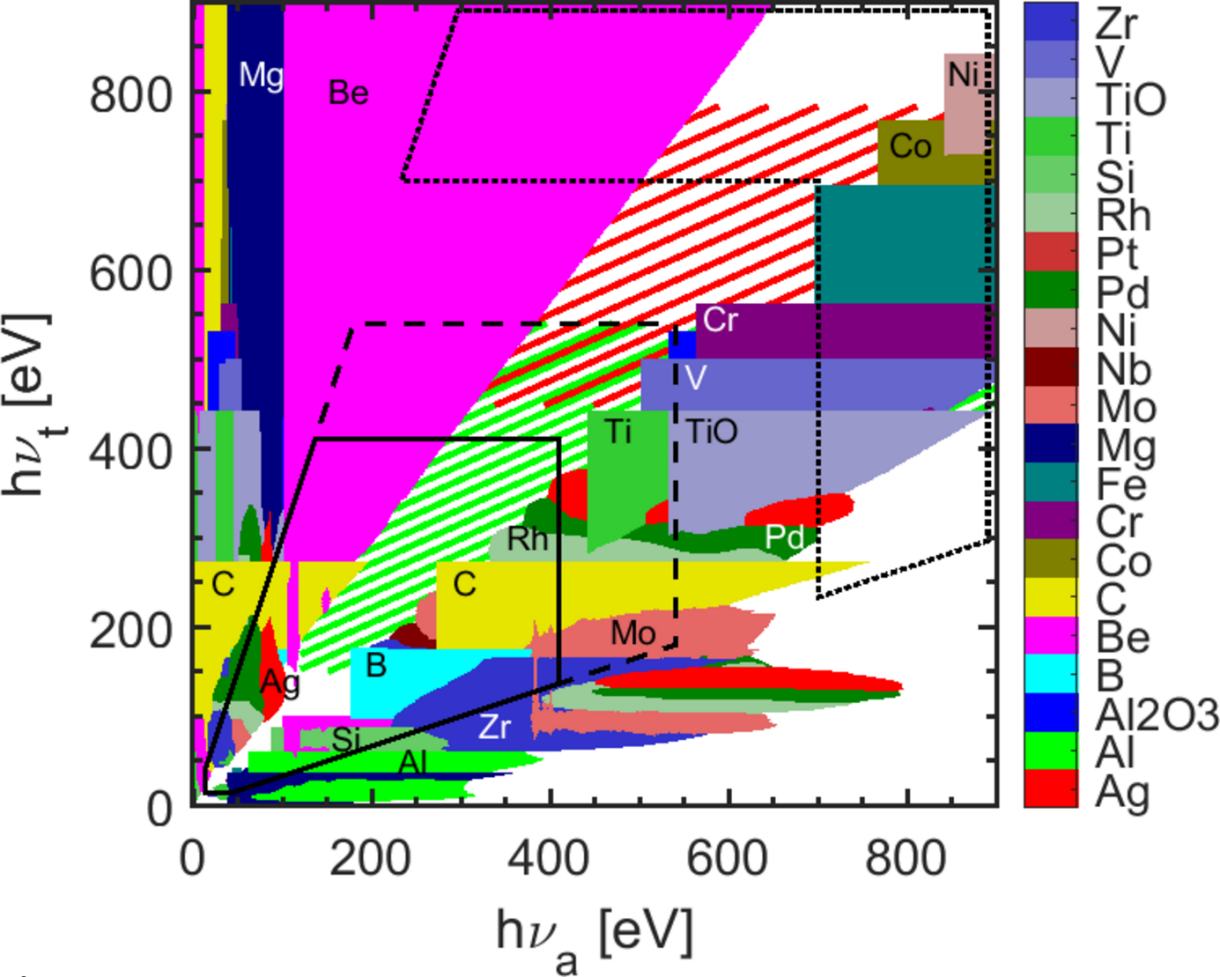
Various filter materials suited for separation of soft X-rays in two-color experiments. Calculated from data provided by the online database CXRO (Henke *et al.*, 1993 ▸). For every entry to the corresponding pair of *h*ν_t_ and *h*ν_a_ the transmission of the transmitted photon energy amounts to *T*_t_(*h*ν_t_, *h*ν_a_) > 2.5% while achieving FR = 100. The hatched areas denote the additional spectral degree of freedom offered by the grating beam path with *T* > 2.5% as taken from Fig. 5 ▸ in first (green) and second order (red). The black marked area shows the expected future limits in two-color operation of FLASH2. The dashed area marks the spectral accessible region when two-color operation is extended to the oxygen *K*-edge. The area included by the dotted line represents the photon energy range in the third harmonic accessible by the afterburner (Tischer *et al.*, 2022 ▸).

The calculations in this paper are based on the transmission data from the CXRO database (Henke *et al.*, 1993 ▸). The transmission of various materials suitable for thin filter fabrication has been calculated. This includes all, to our knowledge, currently commercially available thin filter materials. The minimum manufacturable thickness of the filters, as given by the manufactures (Lebow, Luxel), is taken into account. Fig. 12 ▸ shows the results for FR = 100 and *T*_t_(*h*ν_t_, *h*ν_a_) > 2.5%. For these parameters many applicable filter materials are available in the considered photon energy range. The sharp rectangular features appearing for some elements in this figure originate from the increased absorption at the materials inner shell edges. For a two-color experiment the filtering of both colors is necessary. For transmitting the first color, for example, the corresponding values are *h*ν_t_ = 350 eV and *h*ν_a_ = 200 eV, which results in the spectral area where a beryllium filter is the best choice. For the other beam path it follows that *h*ν_t_ = 200 eV and *h*ν_a_ = 350 eV where carbon is the best suited material for the filtering. In another example, the combination of *h*ν_t_ = 300 eV and *h*ν_a_ = 350 eV yields rhodium as filter. For this case, a Rh filter with about 300 nm thickness is suitable. The corresponding GD of this filter at a bandwidth of Δ*h*ν = 2.0% is GD_2.0%_ ≃ 1 as. The other beam path would need a filter for *h*ν_t_ = 350 eV and *h*ν_a_ = 300 eV, which is shown by the green hatched area. For this combination no filter achieves *T*_t_(*h*ν_t_, *h*ν_a_) > 2.5% for FR = 100. Therefore, the filtering in this case has to be carried out via the grating beam path. A large photon energy separation of the partial beams, *e.g.h*ν_t_ > 1.5 *h*ν_a_ (and its corresponding term *h*ν_t_ < 0.66 *h*ν_a_), can be covered by using various filters almost entirely. For closer photon energies a suitable selection of filters is more difficult, but for *h*ν_t_ < *h*ν_a_ it is still feasible. In the case of photon energies *h*ν_t_ > *h*ν_a_ with only a small energy separation, a gap arises which is caused by the lack of suitable elements. This gap increases with increasing photon energy. Therefore, if photon energies with small energy difference have to be separated, the grating beam path can be employed to solve this issue.

## 5. Summary

A new set-up for the split-and-delay unit serving the beamlines FL23 and FL24 at FLASH is described that enables time-resolved two-color experiments for a wide range of photon energies in the soft X-ray regime. It consists of a combination of filters in both beam paths and a monochromator as an additional beam path. The monochromator will allow both colors within this beam path to be separated. The grating beam path consists of two cylindrical mirrors and two plane VLS gratings operated in first (*m* = 1) and second (*m* = 2) diffraction order. This monochromator covers the spectral range from about *h*ν = 150 eV up to *h*ν = 780 eV with a total transmission of *T* > 2.5% over this entire spectral region. A maximum total transmission of *T* = 18% is provided at a photon energy *h*ν = 353 eV in the first diffraction order and *T* = 13% at a photon energy of *h*ν = 624 eV in the second diffraction order. The resolving power of the monochromator is of the order of 50–200. Consequently, it is comparably low, but sufficient for the separation of both colors in the FEL pulse while preserving the residual induced group delay to GD_0.5%_ < 50 fs. Where the grating beam path is not applicable the additional use of filters extends the parameter range of time-resolved two-color experiments. Since filters are restricted by the available filter materials there is a trade-off between the acceptable filtering ratio and the provided transmission. A transmission of at least *T*_t_ > 2.5% can be achieved while suppressing the other color by a factor of FR = 100 in both the variable and the fixed delay beam path. It should be noted that to a first approximation a filtered beam path does not suffer from group velocity dispersion compared with the grating beam path. With this extension of the present SDU, time-resolved two-color pump–probe experiments will be enabled in which both colors can be chosen with a high degree of freedom.

## Figures and Tables

**Table 1:** Optical properties of the optical elements CM1, CM2, G1, G2 The line density of the VLS gratings is given by the formula *d*(*z*) = *d*_0_ + *d*_1_*z* where *z* = 0 is located in the center of the grating surface.

	CM1	G1	G2	CM2
Type	Cylindrical mirror	Plane VLS grating	Plane VLS grating	Cylindrical mirror
Mirror size (mm)	250 × 25	180 × 25	180 × 25	250 × 25
Clear aperture (mm)	240 × 20	150 × 19	150 × 19	240 × 20
Coating	Ni on Si	Ni on Si	Ni on Si	Ni on Si
Deviation angle (°)	175	175	175	175
Radius (mm)	103857			111827
Entrace arm (mm)	61304			2352
Output arm (mm)	2352			−66008
*d*_0_ (mm^−1^)		200	200	
*d*_1_ (mm^−2^)		0.22	0.22	
Blaze angle (°)		0.50	0.55	

**Table 2:** Distances of the main optical elements in the simulation for the grating beam path

Elements	Distance (mm)
Source–aperture	48104
Aperture–CM1	12750
CM1–G1	560
G1–Slit	1792
Slit–G2	1792
G2–CM2	560
CM2–KB (pre-mirror)	20042
Pre-mirror–KB mirror vertical	1500
KB mirror vertical–KB mirror horizontal	550
KB mirror horizontal–sample	2030
